# Integrated PLS-SEM-Latent Growth Curve Model: A New Conditional Time Invariant Method for Analysing Panel Survey Data

**DOI:** 10.1016/j.mex.2025.103635

**Published:** 2025-09-17

**Authors:** Zulkifli Mohd Ghazali, Wan Yaacob

**Affiliations:** a Mathematical Sciences Studies, Faculty of Computer and Mathematical Sciences, Universiti Teknologi MARA Cawangan Perak, Kampus Tapah, Tapah Road 35400, Perak, Malaysia; b Mathematical Sciences Studies, Faculty of Computer and Mathematical Sciences, Universiti Teknologi MARA, Shah Alam 40450, Selangor, Malaysia; c Institute for Big Data Analytics and Artificial Intelligence (IBDAAI), Kompleks Al-Khawarizmi, Universiti Teknologi MARA, Shah Alam 40450, Selangor, Malaysia

**Keywords:** Panel survey data, Pls-sem, Latent growth curve model, Integrated Model

## Abstract

This study introduces an integrated methodological framework that combines Partial Least Squares Structural Equation Modelling (PLS-SEM) with a conditional time-invariant Latent Growth Curve Model (LGCM) to analyse panel survey data. The integration addresses limitations of existing PLS-SEM approaches in longitudinal research by enabling the simultaneous evaluation of measurement validity, growth trajectories, and predictors of change over time. A disjoint two-stage approach is applied to estimate measurement models and obtain latent scores, which are subsequently used to model growth factors (intercepts and slopes). The proposed method is illustrated using three waves of the Midlife in the United States (MIDUS) study, focusing on life satisfaction and its predictors. Results indicate that psychological well-being and social well-being significantly predict both baseline levels and growth in life satisfaction, while income influences only baseline levels. These findings demonstrate the capacity of the integrated model to disentangle inter-individual differences and developmental patterns in longitudinal data. The findings indicate that:

The integrated PLS-SEM–LGCM framework supports simultaneous analysis of constructs, trajectories, and predictors.

Empirical validation with MIDUS data demonstrates its ability to capture variability in life satisfaction.

Implementation in R through the custom *pls_growth* function enhances reproducibility and accessibility.

## Graphical abstract

**Figure:**
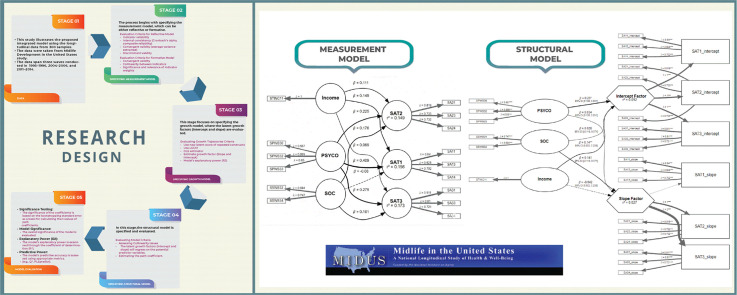

## Specifications table

**Table:** 

**Subject area**	Mathematics and Statistics
**More specific subject area**	Panel Survey Data
**Name of your method**	PLS-SEM Based Model with Conditional Time-Invariant of Latent Growth Curve for Panel Survey Data
**Name and reference of original method**	Original Method: PLS-SEM Higher-order Construct: Disjoint two-stage Reference: Becker, J. M., Klein, K., & Wetzels, M. (2012). Hierarchical Latent Variable Models in PLS-SEM: Guidelines for Using Reflective-Formative Type Models. Long Range Planning, 45(5–6), 359–394. https://doi.org/10.1016/j.lrp.2012.10.001
**Resource availability**	This study used a dataset from samples collected across three waves of the Midlife Development in the United States study (1995–1996, 2004–2006, and 2011–2014) https://www.icpsr.umich.edu/

## Background

Partial Least Squares Structural Equation Modelling (PLS-SEM) is an alternative to the widely used covariance-based SEM (CB-SEM) method for analysing survey data in structural equation modelling techniques. The PLS-SEM approach was initially introduced by [1], and subsequent extensions were suggested by [2]. PLS-SEM has gained popularity due to its ability to estimate complex models, allowing researchers to explore intricate relationships among variables. Recent advancements in model specification and higher-order constructs in PLS-SEM are well-documented in [3], which provides updated guidelines for complex modelling contexts. This method offers flexibility in data requirements and enables the specification of relationships between constructs and indicator variables. Additionally, PLS-SEM is particularly effective in handling non-normal data and small sample sizes, making it suitable for studies with limited data or non-normal distributions [[4], [5], [6]]. Although PLS-SEM has gained popularity, especially in fields like education, marketing, and psychology, its application is limited to cross-sectional survey data ([[7], [8], [9], [10]]). The utilisation of PLS-SEM in panel survey studies, which involves repeated measures over time, is relatively limited. This discrepancy becomes more apparent as there has been an increasing shift from cross-sectional to panel survey studies in recent years, particularly in the context of longitudinal research [[11], [12], [13]]. Several factors contributed to the underutilisation of PLS-SEM in panel survey data, despite the availability of five different approaches in PLS-SEM for analysing panel survey data. Among the approaches are; (i) Pre and Post approach with different construct (model 1), (ii) Path Comparison approach (model 2), (iii) Cross-lagged approach (model 3), (iv) Pre and Post approach with the same construct (model 4), and (v) Evaluation approach (model 5) ([14]).

Although there are five different approaches of PLS-SEM in analysing the panel survey data, these approaches still have limitations and spaces for improvement. The obvious limitation for models 1, 2, 3, and 4 is related to the number of waves for the study, since these approaches are only suitable for two periods of time. In addition, these four models focus on the pre- and post-evaluations and do not measure the evaluations of effects over time. Besides that, model 5 has a limitation in evaluating the growth of trajectory, even though this method is capable of handling studies with more than two periods of time. This model does not have a single structural model to measure the whole changes in the repeated measures simultaneously. In addition, the existing approach or model cannot capture an individual trajectory, the average of the trajectory of the sample or entire group, the evaluation of individual differences in trajectories, and assess the potential incorporation of predictors of individual differences in trajectories. Furthermore, it is also not flexible to simultaneously handle the independent and dependent latent constructs of the same model, allowing for complex representations of growth and correlations of change. These limitations hinder the effective application of PLS-SEM in this specific research context.

Therefore, this study introduces a novel integrated model by combining PLS-SEM with the Latent Growth Curve Model (LGCM) for analysing panel survey data. The integrated approach enhances the capabilities of PLS-SEM to better evaluate growth trajectories, inter-individual differences, and the predictors influencing these changes over time. This study also discusses how to evaluate the measurement model and structural model in PLS-SEM for panel survey data. In the measurement model, the adoption of the standard PLS algorithms to the panel survey data is discussed. Meanwhile, this study also discusses how to develop the structural panel survey equation in the structural model, then evaluates the common latent trajectories, which are the characteristics of the mean trajectory of the entire group, the evaluation of individual differences in trajectories, and the potential incorporation of predictors of individual differences in trajectories.

The remainder of this paper is structured as follows. The Method Details section explains the theoretical framework and the three-stage procedure of the proposed integrated model, including measurement model evaluation, growth modelling, and structural analysis. The Method Validation section presents an empirical illustration using MIDUS panel data and the custom-built *pls_growth* function in R. Finally, the Conclusion section summarizes the key findings, contributions, and limitations of the study, and outlines directions for future research.

## Method details

This section presents an exploration of the foundational concepts and methodologies that underpin this research. The proposed integrated model for panel survey data incorporates elements of both Partial Least Squares Structural Equation Modeling (PLS-SEM) and the Latent Growth Curve Model (LGCM). We begin by explaining the characteristics of panel survey data, the fundamentals of LGCM, and key aspects of PLS-SEM relevant to this study, such as its principles, algorithms, and higher-order models. Finally, we introduce a novel theoretical and conceptual framework specifically designed to apply PLS-SEM in the context of panel survey data.

### Description of panel survey data

The panel survey data is a combination of cross-sectional and time series data types. The panel survey data consists of several units and many points in time. Using the panel survey data, we can observe the trajectories and the factors that influence these changes. The standard notation used for panel survey data is $Y_{it}$, where it is observed for all individuals i=1,2,3,…,N across all time periods t=1,2,3,…,T. Panel survey data is a form of longitudinal study that focuses on capturing changes over time. This type of research often employs the growth equation (Eq. (1) to Eq. (6)) to analyse and understand the patterns of change observed in the data. By tracking the same individuals or groups over multiple points in time, panel surveys provide valuable insights into how various factors influence and shape these changes.

### Latent growth curve model (LGCM) for panel survey data

The latent growth curve model (LGCM) is formed from the development of latent variable modelling and factor analysis [[15], [16], [17]]. Applying LGCM to panel survey data helps researchers understand dynamic processes. It reveals the underlying changes and transformations within a population. The LGCM has become widely used in behavioural sciences research. It is applied in various field, including social behavioural research, psychology (encompassing clinical, developmental, and educational research), studies on learning and memory, as well as research on personality [[18], [19], [20], [21], [22], [23]]. This method has an advantage in the investigation of inter-individual differences in change over time, as it allows investigation the antecedents and consequences of change [24]. LGCM also provides group-level statistics, such as the mean growth rate and mean intercept, and can test hypotheses of specific trajectories. Additionally, it allows the incorporation of time-invariant covariates. The basic LGCM can be described as an unconditional growth model. The general unconditional latent growth curve model is as follows.

(1) $$y_{it}=\alpha_{i}+\lambda_{t}\beta_{i}+\epsilon_{it}$$

where $y_{it}$ is the value of the trajectory variable y for the ith case at time t, $\alpha_{i}$ is the random intercept for case i, and $\beta_{i}$ is the random slope for case i. From Eq. (1), the random intercepts and slopes equations are as follows.

(2) $$\alpha_{i}=\mu_{\alpha }+\zeta_{\alpha i}$$

(3) $$\beta_{i}=\mu_{\beta }+\zeta_{\beta i}$$

where $\mu_{\alpha }$ and $\mu_{\beta }$ are the mean intercepts and mean slopes.

### Conditional model

To incorporate the potential independent variables that predict the growth factor, the conditional LGCM should be used. The conditional model starts with the same equation as the unconditional model, Eq. (1).

(4) $$y_{it}=\alpha_{i}+\lambda_{t}\beta_{i}+\epsilon_{it}$$

However, in the level 2 equations, the equations for random intercepts and slopes ((2), (3)) are modified to incorporate the potential predictor variables. The conditional random intercepts and slopes equations are as follows.

(5) $$\alpha_{i}=\mu_{\alpha }+\gamma_{\alpha 1}x_{1i}+\zeta_{\alpha i}$$

(6) $$\beta_{i}=\mu_{\beta }+\gamma_{\beta 1}x_{1i}+\zeta_{\beta i}$$

where $\mu_{\alpha }$ and $\mu_{\beta }$ are the mean intercepts and mean slopes when $x_{1i}$ is zero. The $x_{1i}$ is covariate or predictor of the random intercepts and slopes, while $\gamma_{\alpha 1}$ and $\gamma_{\beta 1}$ are the covariate coefficients for $x_{1i}$ in the random intercept and slope equations. This predictor is known as time-invariant covariate which does not vary over time.

### Factor loadings

The factor loading ($\lambda_{t}$) is the time factor that can allow the incorporation of linear and curvilinear trajectories. There are two types of loading, which are fixed loading (represents a linear trajectory) and free loading (represents a curvilinear trajectory). In the linear trajectory, the model uses a fixed loading where $\lambda_{t}$ is equal to T−1 for all T. In contrast, to estimate the curvilinear trajectory, [25] suggested the first loading $\lambda_{1}=0$ and the last loading $\lambda_{T}=1$. Then, all other loadings between the first and last are freely estimated. However, in this study, we focus on the linear trajectory only.

### Principles of pls-sem

**Fig. 1:**
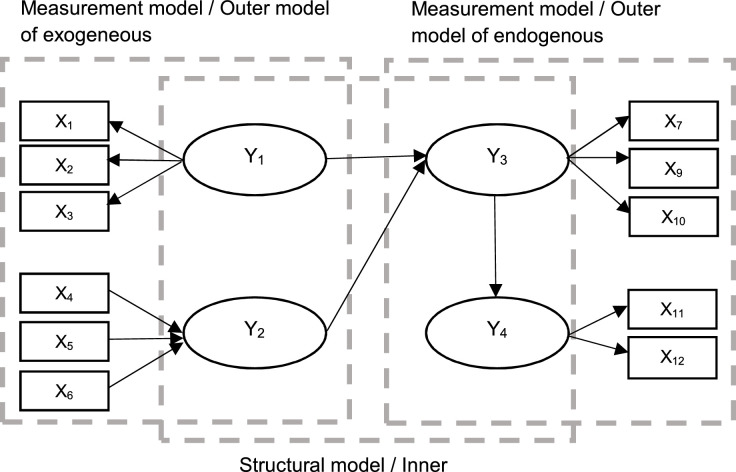
The measurement and structural models of the PLS-SEM.

Partial Least Squares Structural Equation Modelling (PLS-SEM) is a widely utilized statistical technique in survey studies. It offers the advantage of examining both observed and unobserved variables within a research framework. PLS-SEM consists of two main components which are measurement model and structural model. The measurement model establishes the relationship between constructs and their indicators, enabling researchers to assess the extent to which the indicators accurately measure the underlying constructs. On the other hand, a structural model represents the relationships between each of the constructs, enabling the exploration of direct and indirect effects among the variables of interest. The measurement and structural models are illustrated in Fig. 1.

### PLS-SEM algorithm

**Fig. 2:**
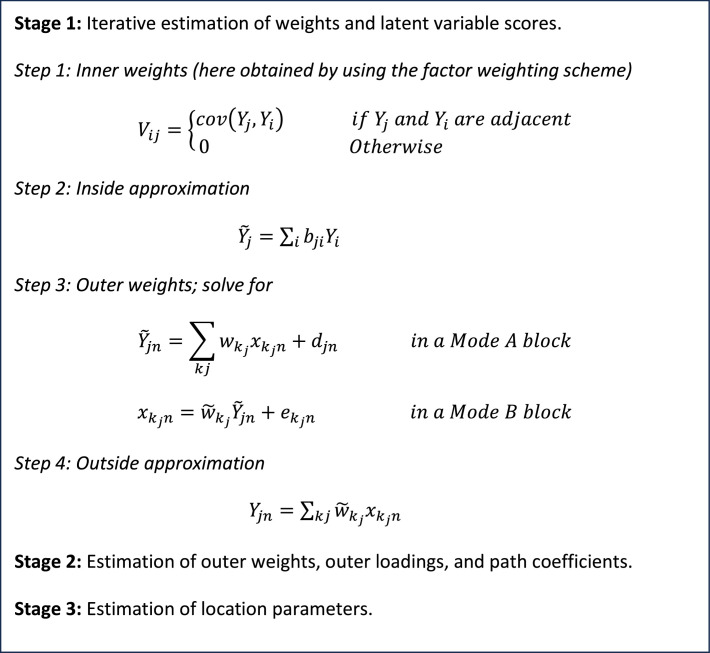
Procedure in PLS-SEM Algorithm for Measurement Model.

The PLS-SEM algorithm involves a three-stage approach that belongs to the family of least squares algorithms as illustrated in Fig. 2. In the initialization stage, preliminary latent variable scores are calculated using unit weights for all indicators. These scores provide a starting estimate that is refined in later stages of the algorithm. To compute these scores, the algorithm typically uses unit weights (starting with 1) for all indicators in the measurement models. The initialization stage is important because it can affect the convergence and stability of the algorithm. Therefore, it is recommended to use a reliable initialization method, such as the centroid method, the factor-based method or path weighting scheme, to obtain more accurate estimates of the latent variables. The second stage is the outer model estimation stage, where the measurement model is estimated by regressing the observed variables on their respective latent variables. The third stage is the inner model estimation stage, where the structural model is estimated by regressing the endogenous latent variables on the exogenous latent variables.

### Higher order construct

In the field of survey studies, evaluating higher-order constructs poses a methodological challenge, as these latent variables cannot be directly observed. Various methods are employed to address this challenge and to gain insight into the underlying relationships within the data. Within the realm of Partial Least Squares Structural Equation Modelling (PLS-SEM), two principal approaches are commonly utilized. The repeated indicators approach uses multiple indicators or observable variables to measure the higher-order construct. This provides a direct and observable representation of the latent variable ([2,26]).

Conversely, the two-stage approach takes a more indirect route, introducing an additional layer to the analysis. Within the two-stage approach, researchers have proposed specific methods to navigate the intricacies of evaluating higher-order constructs. Two noteworthy methods are the embedded two-stage approach [27] and the disjoint two-stage approach [[27], [28], [29]]. In the context of our study, we adapted the disjoint two-stage approach. The disjoint two-stage approach involves initially assessing the lower-order component of the higher-order construct. In the first stage, the lower-order component is evaluated, and its latent score is calculated and saved. Then the saved score is used to assess the higher-order construct. Table 1 shows the summary of measurement specifications of the lower-order component and higher-order construct for the reflective-reflective and formative-reflective models as illustrated by Sarstedt et al. [30].

**Table 1:** Measurement Specification of the Higher-order Construct.

### Proposed theoretical and conceptual framework of pls-sem panel survey data

**Fig. 3:**
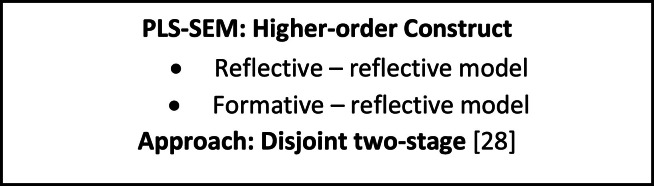
Theoretical framework of PLS-SEM with higher-order constructs.

Panel survey data offers a dynamic perspective on social phenomena by capturing both the breadth of cross-sectional data and the depth of time series data. In this study, the theoretical framework (Fig. 3) centres on employing Partial Least Squares Structural Equation Modelling (PLS-SEM) with a higher–order model, demonstrating its aptness for panel survey data. The comprehensive framework includes reflective–reflective and formative–reflective models, capturing the complexities of relationships among latent constructs. The estimation of this intricate model is facilitated by the disjoint two-stage approach, a methodical strategy that enhances precision in the analysis. This approach allows separate estimation of the lower-order components and the higher-order construct. It ensures a careful examination of individual constructs and their interrelationships within the specified models.

PLS-SEM is a powerful statistical method that excels in analysing complex relationships among latent variables. The adaptability of PLS-SEM is a cornerstone of its effectiveness in panel survey data analysis. PLS-SEM not only facilitates the evaluation of indicators for constructs but also flexibly empowers researchers to model higher-order constructs. This unique flexibility, making it well-suited for panel survey data analysis, allows for a holistic examination of the indicators for each repeated construct. In conjunction with this analytical framework, we incorporate Latent Growth Curve Models (LGCM), a class of statistical models designed for analysing the trajectories (individual and overall) of latent variables over time. Strengthening this approach, the conditional model in LGCM goes beyond merely describing the trajectories as it allows researchers to assess potential predictor variables and examine the factors influencing those trajectories.

**Fig. 4:**
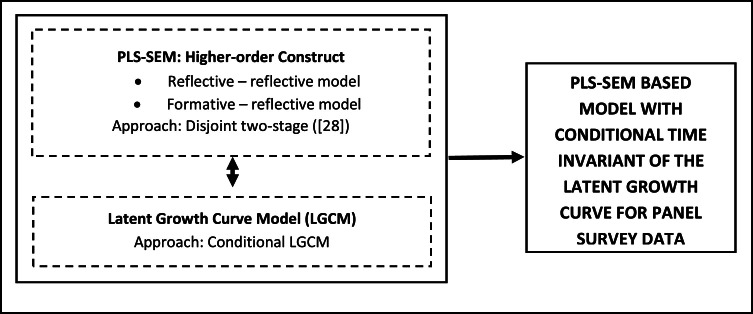
Conceptual framework integrating PLS-SEM and LGCM for analysing panel survey data.

The integration of PLS-SEM with conditional LGCM presents a powerful strategy for advancing panel survey data analysis, offering a range of benefits that collectively enhance the depth and precision of longitudinal research. PLS-SEM algorithm evaluates the reliability and validity of the indicators for each repeated construct, ensuring a robust assessment of the measurement model. Additionally, LGCM contributes to the analysis by evaluating not only the trajectories but also examining the potential predictor variables that may influence the observed trajectories. This new approach establishes a comprehensive framework (Fig. 4) for understanding the complexities of panel survey data, encompassing measurement reliability and validity, trajectories, and exploring influential factors within the longitudinal context.

### Integrated model specification

**Fig. 5:**
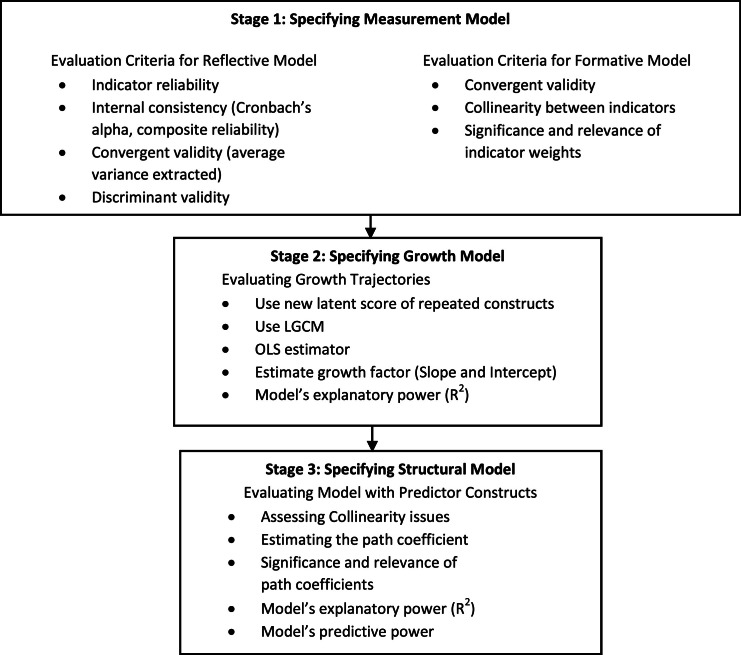
Flow in the Integrated Model.

This integrated model has adapted a disjoint two-stage approach strategy for estimating the model, consisting of three sequential stages (Fig. 5). In stage 1, the process begins with specifying the measurement model, which can be either reflective or formative. For reflective models, evaluation criteria include indicator reliability, internal consistency (assessed using Cronbach's alpha and composite reliability), convergent validity (measured through average variance extracted), and discriminant validity (ensuring constructs that are supposed to be unrelated are distinct from each other). For formative models, the criteria include convergent validity (assessing the relationship between formative indicators and their latent construct), collinearity between indicators (ensuring that indicators are not excessively correlated), and the significance and relevance of indicator weights (evaluating the importance of each indicator in forming the construct). These criteria align with recent guidelines recommending Confirmatory Composite Analysis (CCA) for assessing reflective measurement models in PLS-SEM [31].

Moving to the second stage, this integrated model focuses on specifying the growth model, where the latent growth factors (intercept and slope) are evaluated. The Ordinary Least Squares (OLS) estimator is used to estimate the model parameters, and the model's explanatory power $\left(R^{2}\right)$ is assessed to determine how well the model explains the variability in the outcome.

In stage 3, the structural model is specified and evaluated. This includes assessing collinearity issues to ensure that predictor variables are not highly collinear, estimating path coefficients to calculate the strength and direction of relationships between constructs (predictors and latent growth factors), and determining the significance and relevance of these path coefficients. The model's explanatory power $\left(R^{2}\right)$ is re-evaluated to see how well it explains the variance in the latent growth factor, and the model's predictive power is assessed to determine its ability to predict future observations. Finally, bootstrapping is employed to evaluate the statistical significance of the model parameters. This resampling method involves repeatedly sampling from the data with replacement to create a distribution of the parameter estimates, which helps in evaluating the precision of the estimates and testing the stability and reliability of the model.

### Integrated model evaluation

This section discusses the criteria and the rules of thumb for each stage as illustrated in Fig. 5.

Stage 1: Specifying Measurement Model

### Reflective measurement model

**Fig. 6:**
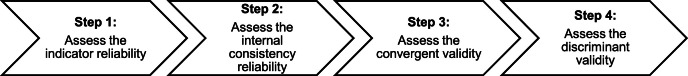
Reflective measurement model evaluation procedure.

Fig. 6 shows the reflective measurement model evaluation procedure. Reflective measurement models are evaluated by looking at measures' internal consistency dependability and their level of reliability (indicator reliability). The average variance extracted (AVE) is used in the validity assessment process to target each measure's convergent validity. Furthermore, the discriminant validity of a reflectively measured construct is assessed relative to other construct measures in the same model using the heterotrait-monotrait (HTMT) ratio of correlations.

In step 1, reflective indicator loadings are used to assess the relationship between latent constructs and their observed indicators. The threshold for reflective indicator loadings is set at ≥ 0.708. This criterion ensures that each indicator sufficiently explains the variance of the underlying construct. A loading of 0.708 implies that approximately 50 % of the variance in the indicator is explained by the construct (since $0.708^{2}\approx 0.50$). Indicators with loadings below this threshold may need to be reevaluated or potentially excluded to maintain the construct’s validity.

Moving to step 2, internal consistency reliability measures the extent to which indicators of a construct are correlated, providing a gauge of the construct’s overall reliability. Cronbach’s alpha and composite reliability (rho_c_) are the primary metrics for this criterion. Cronbach’s alpha is considered the lower bound, while rho_c_ serves as the upper bound for internal consistency reliability. The reliability coefficient rho_A_, typically lying between these bounds, offers a balanced representation of internal consistency. The thresholds for internal consistency reliability are a minimum of 0.70 (or 0.60 in exploratory research), a maximum of 0.95 to avoid indicator redundancy and compromised content validity, and a recommended range of 0.80 to 0.90. These thresholds ensure a balance between sufficient internal consistency by avoiding excessive redundancy among indicators.

Step 3 involves convergent validity assessing whether indicators of a construct that are theoretically related are indeed related in practice. The average variance extracted (AVE) is the metric used to evaluate convergent validity, with a threshold of AVE ≥ 0.50. An AVE of 0.50 or higher indicates that at least 50 % of the variance in the indicators is explained by the construct, signifying adequate convergent validity.

Finally, discriminant validity examines whether constructs that are supposed to be unrelated are indeed distinct from one another. The heterotrait-monotrait ratio of correlations (HTMT) is employed to evaluate discriminant validity. The thresholds for HTMT are less than 0.90 for conceptually similar constructs and less than 0.85 for conceptually different constructs. Additionally, it is important to test if the HTMT value is significantly lower than the specified threshold, ensuring that constructs are not only theoretically but also empirically distinct.

### Formative measurement model

**Fig. 7:**
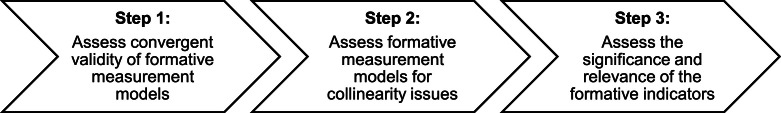
Formative measurement model evaluation procedure.

Fig. 7 shows the formative measurement model evaluation procedure. Formative measurement models are evaluated using several criteria, including convergent validity, collinearity, the statistical significance of indicator weights, and the relevance of indicators.

Firstly, the convergent validity in formative measurement models is assessed through redundancy analysis. This involves examining the correlation between the formative construct and a reflective (or single item) measurement of the same concept. The threshold for adequate convergent validity is a correlation of ≥ 0.708. This high correlation ensures that the formative construct adequately captures the same concept as the reflective measure, indicating that the indicators collectively represent the construct well.

Moving to the second step, the collinearity refers to the degree to which indicators are correlated with each other. High collinearity can pose significant issues in formative measurement models, potentially distorting the results and interpretations. The variance inflation factor (VIF) is used to assess collinearity, with critical collinearity issues likely occurring if VIF is 5 or greater, collinearity issues usually being uncritical if VIF is between 3 and 5, and collinearity not being problematic if VIF is less than 3. Maintaining VIF values below these thresholds is crucial to ensure that collinearity does not adversely affect the model's validity and reliability.

Finally, the statistical significance of indicator weights is a crucial aspect of formative measurement models. The t-values of indicator weights should be greater than 2.576(α=0.01),1.960(α=0.05),or1.645(α=0.10) respectively, for two-tailed tests. Furthermore, the 95 % percentile confidence interval (α=0.05) should not include zero. These criteria ensure that the weights of the indicators are statistically significant, confirming their contribution to the construct. Indicators with significant weights contribute meaningfully to the construct, and the larger the significant indicator weight, the higher the relative contribution of that indicator to the construct. This relevance indicates that certain indicators play a more crucial role in defining the construct, and their significance should be carefully considered in the model assessment. Even if an indicator's weight is not statistically significant, it can still be relevant if it has a loading of 0.50 or greater and is statistically significant. Such indicators are considered important for the construct as they represent substantial aspects of the concept being measured. This criterion ensures that indicators with moderate but significant contributions are not overlooked in the assessment process.

Besides evaluating the measurement model, the latent scores for each construct are also estimated and saved for subsequent analyses.

Stage 2: Specifying Growth Model

Building on the latent scores estimated in Stage 1, the second stage focuses on specifying the growth model, where the latent growth factors (intercept and slope) are evaluated. The unconditional latent growth curve model (Eq. (1) to Eq. (3)) is established, which involves only repeated constructs. To estimate this model, key parameters include the individual-specific intercepts $\left({\hat{\alpha }}_{i}\right)$ and slopes $\left({\hat{\beta }}_{i}\right)$, their population means $\left(\mu_{\alpha },\;\mu_{\beta }\right)$, residual variance (Var(ϵ)) and the variances of the random effects $\left(\psi_{\alpha \alpha \;},\;\psi_{\beta \beta }\right)$. This model assumes a linear growth trajectory, where change in the repeated constructs occurs at a constant rate over time, and equally spaced time scores are applied using fixed loadings $\lambda_{t}=t-1$. These assumptions correspond to a conventional linear latent growth model in which a single intercept and slope represent each individual's trajectory.

In addition, the model assumes that residuals $\left(\epsilon_{it}\right)$ are independently and identically distributed with constant variance and zero mean $\left(\epsilon_{it}\;∼N\left(0,\;\sigma^{2}\right)\right)$, and that the random intercepts and slopes are drawn from a multivariate distribution. However, because the latent scores used in this stage are obtained from the PLS-SEM algorithm using Ordinary Least Squares (OLS), the estimation does not require multivariate normality. This feature aligns with the distribution-free nature of PLS-SEM and supports the use of non-parametric bootstrapping in the final structural model stage. Although the linear trajectory provides a parsimonious and interpretable framework, future studies should extend the model to accommodate non-linear change patterns. For example, quadratic growth models capture acceleration or deceleration trends, and piecewise models allow examination of segmented growth phases with varying slopes. These potential extensions enhance the model’s flexibility and provide a more nuanced representation of individual developmental trajectories over time.

Following the measurement model evaluation in Stage 1, the standardized latent scores of the repeated constructs are extracted. These scores represent each individual’s estimated position on the latent variable at each time point. To prepare these scores for trajectory estimation, reverse standardization is applied. The standardized scores are multiplied by the standard deviation and then added to the mean of the original observed construct values. This step restores the latent scores to the original scale of the construct.

Next, the latent growth factors ($\alpha_{i}$ and $\beta_{i}$) are estimated using ordinary least square estimator (OLS). This is done by treating the reverse-standardized latent scores as outcomes and using time scores $\lambda_{t}$ as predictors. This can be expressed in the (7), (8). This estimation produces the intercept and slope for individual i.

(7) $${\hat{\beta }}_{i}=\frac{\sum_{t=1}^{T}\left(\lambda_{t}-\bar{\lambda }\right)\left(y_{it}-{\bar{y}}_{i}\right)}{\sum_{t=1}^{T}{\left(\lambda_{t}-\bar{\lambda }\right)}^{2}}$$

(8) $${\hat{\alpha }}_{i}=\bar{y_{i}}-{\hat{\beta }}_{i}\bar{\lambda }$$

The resulting coefficients $\alpha_{i}$ and $\beta_{i}$ represent the individual’s initial status and rate of change, respectively. The mean trajectory for the entire sample is estimated by averaging these parameters as shown in (9), (10).

(9) $${\hat{\mu }}_{\alpha }=\frac{\sum_{i=1}^{N}{\hat{\alpha }}_{i}}{N}$$

(10) $${\hat{\mu }}_{\beta }=\frac{\sum_{i=1}^{N}{\hat{\beta }}_{i}}{N}$$

Next, to estimate the differences of trajectories between the individual, the variance of intercept and slope is assessed. The equation for assessing the variance is shown in (13), (14).

(11) $$var\left(\epsilon \right)=\frac{\sum_{i=1}^{N}var\left(\epsilon_{i}\right)}{N}$$

(12) $$var\left(\epsilon_{i}\right)=\frac{\sum_{t=1}^{T}e_{it}^{2}}{T-2}$$

(13) $${\hat{\psi }}_{\alpha \alpha }=var\left(\hat{\alpha }\right)-\frac{var\left(\epsilon \right)\sum_{t=1}^{T}\lambda_{t}^{2}}{\left[\sum_{t=1}^{T}{\left(\lambda_{t}-\bar{\lambda }\right)}^{2}\right]T}$$

(14) $${\hat{\psi }}_{\beta \beta }=var\left(\hat{\beta }\right)-\frac{var\left(\epsilon \right)}{\sum_{t=1}^{T}{\left(\lambda_{t}-\bar{\lambda }\right)}^{2}}$$

Then, the $R^{2}$ is used to evaluate the model fit. $R^{2}$ values for each regression provide information on the closeness of the linear trajectory to the data points observed. This second stage thus translates PLS-estimated latent scores into individual growth trajectories while retaining robustness to non-normal data. The estimated growth parameters are subsequently analyzed in Stage 3 to identify significant predictors within the structural model using the PLS-SEM framework.

Stage 3: Specifying Structural Model

**Fig. 8:**
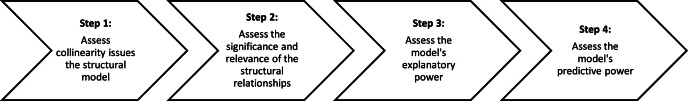
Structural model evaluation procedure.

In the third stage, the structural model is evaluated based on the PLS-SEM framework as illustrated in Fig. 8. This stage involves evaluating potential predictor variables towards the trajectories by employing the OLS estimator. The latent growth factors (intercept and slope) will regress on the potential predictor variables. The coefficients are estimated, and the structural model must assess the potential of collinearity issues before proceeding to the subsequent tests, which are to examine the significance of these potential predictor variables. The significance of the coefficients is based on the bootstrapping standard error as a basis for calculating the t-values of path coefficients. This stage plays a crucial role in understanding the factors influencing the trajectories. Finally, the overall significance of the model is evaluated using the $R^{2}\;$value, which offers a comprehensive perspective on the explanatory power of the entire integrated model.

The collective findings from these three stages contribute to a nuanced understanding of the evaluation of the measurement model, the mean trajectory of the entire group, the evaluation of individual differences in trajectories, and the potential incorporation of predictors of individual differences in trajectories.

### Analysis using R software

The proposed integrated model is implemented in R using a custom function, “***pls_growth****”*, developed specifically for this framework. The function enables modelling growth trajectories within the PLS-SEM context, supported by the SEMinR package. Table 2 shows how to install and begin using the PLSgrowth package. Additionally, various functions from the SEMinR package, such as *construct, relationships, and bootstrap_model,* were used to define the measurement model, estimate the structural model, and perform bootstrapping analysis, respectively. Table 3 illustrates the use of the pls_growth function and several functions in the SEMinR package.

**Table 2:** Installation of the PLSgrowth package.

Install the devtools package if not already installed.	*install.packages("devtools")*
Install PLSgrowth from GitHub.	*devtools::install_github("MrZMG/PLSgrowth")*
Load the package into your R session.	*library(PLSgrowth)*

**Table 3:** Summary of R code for the proposed integrated model.

Define the measurement and structural model using constructs and relationships function from SEMinR package.	sat_mm <- constructs( reflective("SAT1", multi_items("SA1", c(1, 3, 4))), reflective("SAT2", multi_items("SA2", c(1, 3, 4))), reflective("SAT3", multi_items("SA3", c(1, 3, 4))), reflective("PSYCO", multi_items("SPWB3", c(2, 3, 6))), reflective("SOC", multi_items("SSWB3", c(2, 4))), reflective("Income", single_item("STINC11")), higher_composite("GROWTH", c("SAT1", "SAT2", "SAT3"))) sat_sm <- relationships( paths(from = "PSYCO", to = "GROWTH"), paths(from = "Income", to = "GROWTH"), paths(from = "SOC", to = "GROWTH"))
Estimate the measurement, growth and structural model using ‘*pls_growth*’ function.	sat_pls <- pls_growth( data = data, measurement_model = sat_mm, structural_model = sat_sm, missing = mean_replacement, include_tvcs = FALSE, missing_value = FALSE)
Bootstrap for significance and relevance of path coefficients.	boot <- bootstrap_model(seminr_model = sat_pls, seed = 123)

## Method validation

This section illustrates the proposed integrated model using data from 300 samples collected across three waves of the Midlife Development in the United States study (1995–1996, 2004–2006, and 2011–2014). In this empirical example, we employed a reflective-reflective model, which focuses on how indicators reflect the underlying constructs, as opposed to the formative-reflective model. The repeated construct in this model is life satisfaction (SAT1, SAT2 and SAT3), measured over time. Additionally, the model includes three predictor constructs that serve as time-invariant covariates, namely psychological well-being (PSYCO), social well-being (SOC), and household income (INCOME). This integrated approach aims to examine the trajectories or rate of change in life satisfaction and how these predictors influence life satisfaction across the different waves of the study.

**Fig. 9:**
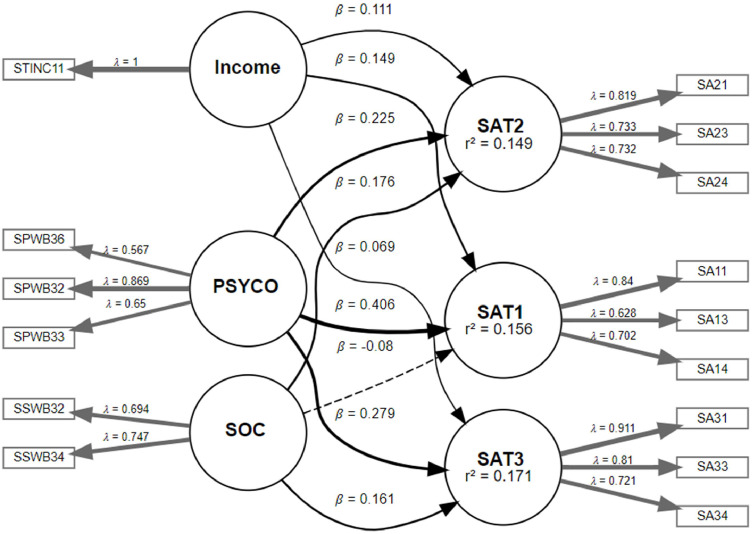
Evaluation of the measurement model in the first stage.

In the first stage, we create the initial model to evaluate the measurement model by connecting all the latent exogenous variables to the lower-order component known as latent repeated constructs as displayed in Fig. 9. Then, the model needs to satisfy all the criteria mentioned earlier in Fig. 6, and the results are shown in Table 4, Table 5. The latent score for the repeated constructs is also saved, which will be used in the next stage of the analysis to further explore their trajectories on life satisfaction over time.

**Table 4:** Reliability and validity statistics of constructs.

	Cronbach’s α	$\rho_{A}$	Composite reliability $\rho_{c}$	Average variance extracted (AVE)
PSYCO	0.743	0.776	0.743	0.500
SOC	0.682	0.685	0.683	0.519
SAT1	0.766	0.785	0.770	0.531
SAT2	0.804	0.809	0.806	0.581
SAT3	0.854	0.867	0.857	0.669

**Table 5:** Discriminant validity assessment using the HTMT criterion.

	PSYCO	Income	SOC	SAT1	SAT2	SAT3
PSYCO						
Income	0.051					
SOC	0.704	0.018				
SAT1	0.355	0.17	0.191			
SAT2	0.342	0.12	0.324	0.661		
SAT3	0.395	0.077	0.346	0.478	0.638	

**Fig. 10:**
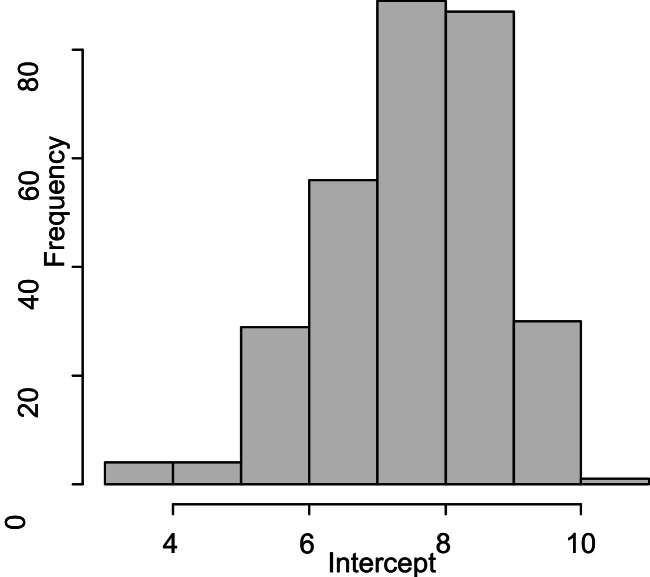
Histogram of intercept of life satisfaction.

In the second stage, latent growth estimates were derived by regressing repeated measures of life satisfaction over time. This approach enabled the estimation of individual trajectories using two growth parameters, namely the intercept, which represents the initial status, and the slope, which captures the rate of change over time. The distribution of intercepts, as visualised in Fig. 10, shows that individuals began with a high level of life satisfaction. The mean intercept in Table 6 was 7.5228 (SE = 0.0717, 95 % CI [7.3823, 7.6632]), indicating that the typical participant perceived themselves as moderately to highly satisfied with life at the initial measurement occasion. However, the variance in intercepts, recorded at 0.8642, shows considerable interindividual variability in baseline levels of life satisfaction. This means that while some participants reported relatively low initial satisfaction, others reported much higher values.

**Table 6:** Parameter estimate for the growth model.

Parameter	Estimate
var(ϵ)	0.8327
$\mu_{\alpha }$	7.5228 (0.0717)
$\mu_{\beta }$	−0.2061(0.0472)
$\psi_{\alpha \alpha }$	0.8462
$\psi_{\beta \beta }$	0.2530
$CI_{\mu_{\alpha }}$	(7.3823, 7.6632)
$CI_{\mu_{\beta }}$	(−0.2987, −0.1135)

**Fig. 11:**
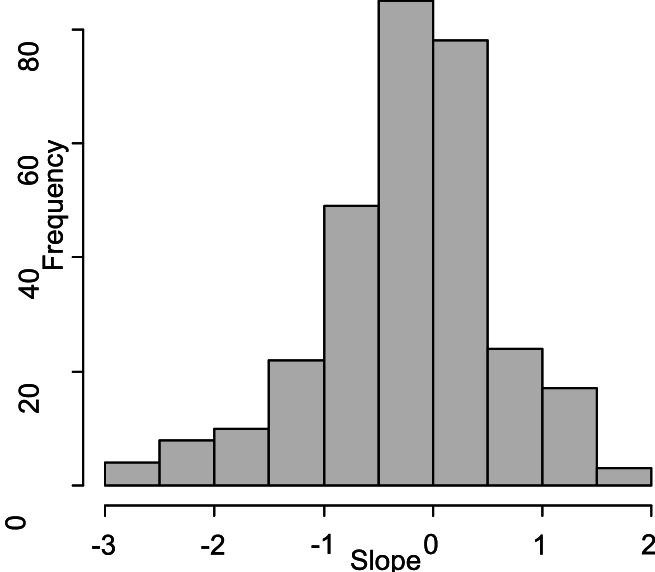
. Histogram of slope of life satisfaction.

Additionally, the slope distribution, presented in Fig. 11, reveals more pronounced heterogeneity in the direction and rate of change. The mean slope in Table 6 was estimated at −0.2061 (SE = 0.0472, 95 % CI [−0.2987, −0.1135]), indicating that participants experienced a decline in life satisfaction throughout the study period. The variance in slope values, which reached 0.2530, indicates that while some participants exhibited a downward trend, others either maintained stability or showed improvement. This is evident from the presence of both positive and negative slopes in the histogram.

Such interindividual variability is a key advantage of the latent growth modelling approach, as it emphasises that not all participants follow the same developmental pattern. The combination of declining, stable, and improving trajectories indicates that a variety of personal, social, and environmental factors influence life satisfaction during midlife. These findings demonstrate diverse developmental experiences across individuals.

Regarding theoretical relevance, the overall decline in life satisfaction can be understood through lifespan development theories, which suggest that midlife is often marked by increasing life demands, transitions, and role strain, all of which could negatively impact subjective well-being. Simultaneously, the significant variability in growth slopes indicates the influence of buffering or enhancing factors, such as resilience, coping resources, or social support. This variability emphasises the practical importance of identifying conditions that promote more positive developmental pathways in life satisfaction.

**Fig. 12:**
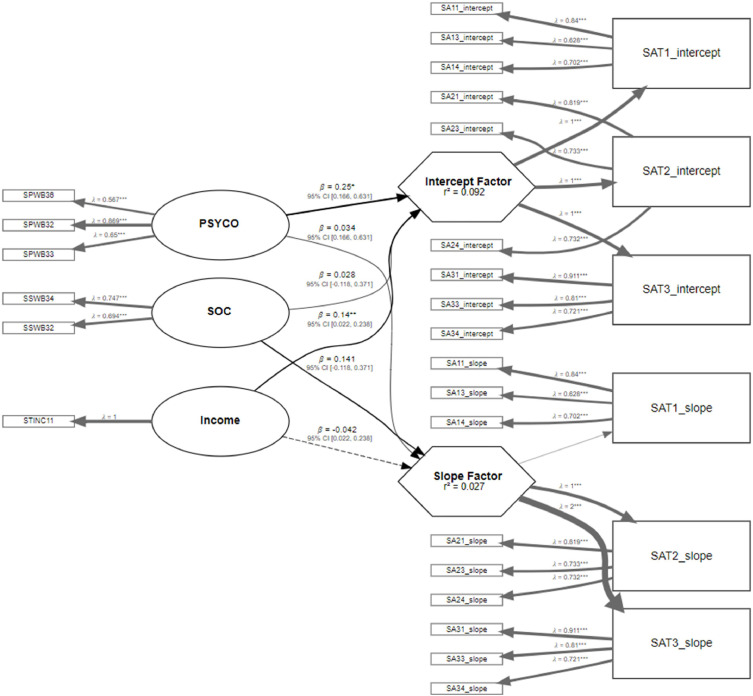
Bootstrapping of Integrated PLS-SEM with LGCM results.

Finally, the structural model is evaluated by addressing the potential predictor construct that influences the trajectories. This evaluation is made by regressing the slope and intercept over the potential predictor construct. Then, the bootstrap method is employed to assess the significance of the path coefficients based on t-values or confidence intervals, as shown in Fig. 12. Fig. 12 illustrates the final stage of the integrated model, where the structural relationships between the predictor constructs (psychological well-being, social well-being, and income) and the two latent growth parameters (intercept and slope of life satisfaction) are examined. The path diagram highlights the standardized path coefficients and their corresponding levels of significance obtained via bootstrapping. In this empirical application, psychological well-being (PSYCO) exhibited a substantial and statistically significant positive effect on the intercept, indicating that individuals with higher baseline psychological well-being also reported higher initial life satisfaction. Similarly, PSYCO demonstrated a small but significant negative effect on the slope, suggesting a buffering role in the decline of life satisfaction over time. Meanwhile, social well-being (SOC) showed a significant positive influence on both the intercept and slope, implying that higher levels of perceived social support are associated with initially higher life satisfaction and a more stable or less negative trajectory across time. Lastly, household income was found to be significantly associated only with the intercept, but not the slope, indicating its role in shaping the baseline level of life satisfaction rather than the rate of change. Overall, these findings validate the capacity of the proposed integrated model to disentangle the differential effects of predictors on baseline status and developmental trajectories, offering meaningful insights into the dynamic processes of well-being over time.

## Conclusion

The integration of Partial Least Squares Structural Equation Modelling (PLS-SEM) with the Latent Growth Curve Model (LGCM) proposed in this study offers a novel and robust approach for analysing panel survey data. This integrated model overcomes several limitations of traditional PLS-SEM, particularly in longitudinal research, by enabling the evaluation of growth trajectories, individual differences, and potential predictors over time. The use of the disjoint two-stage approach for higher-order constructs, combined with the flexibility of PLS-SEM and the dynamic analysis capabilities of LGCM, provides a comprehensive framework for examining longitudinal data.

Compared to conventional latent growth curve modelling based on covariance-based SEM, the proposed model offers several methodological advantages. Traditional LGCM assumes multivariate normality and typically focuses on reflective constructs, which limit flexibility in handling more complex model structures. In contrast, this integrated approach capitalises on the distribution-free nature of PLS-SEM, allowing both reflective and formative measurement models, and is well-suited for small to moderate sample sizes and non-normal data. By estimating latent scores through PLS in Stage 1 and using these to model growth trajectories in Stage 2, followed by structural evaluation in Stage 3, the model supports a modular yet unified analysis of measurement, growth, and prediction components. This makes it a practical and flexible alternative for researchers dealing with panel survey data. Several key contributions that emphasise the novelty of this approach are shown in Table 7.

**Table 7:** Elaboration on the contributions to this study.

Methodological Innovation	The combination of PLS-SEM with LGCM is a novel advancement, allowing for a more thorough analysis of the reliability and validity of construct and growth trajectories within the same model while capturing complex relationships and changes over time.
Extension to Longitudinal Data	By adapting PLS-SEM for panel survey data, this study expands the method’s applicability beyond cross-sectional analyses. This method enables longitudinal modelling that accommodates growth trajectories and latent constructs simultaneously and evaluates the factors that influence trajectories.
Enhanced Analytical Framework	The use of the disjoint two-stage approach for higher-order constructs within the integrated model adds a methodological contribution. The detailed discussion of measurement model specifications, growth model estimation, and structural modelling in a longitudinal context offers greater precision and flexibility in modelling complex relationships in panel data.
Practical Implementation in R	The development of the custom ***pls_growth*** function in R, alongside the SEMinR package, facilitates the application of this integrated model, making it accessible to researchers and practitioners who work with longitudinal data.

This new approach not only improves the reliability and validity of panel data analysis but also provides deeper insights into individual trajectories and the factors that influence changes over time. As a result, this study offers a valuable contribution to both the methodological literature and practical applications in the fields of behavioural sciences, education, and social research, where understanding dynamic processes over time is critical.

## Limitations

• This study focuses on linear growth models to examine changes over time. It does not consider non-linear or polynomial models, which better capture complex developmental patterns such as acceleration or deceleration in certain variables.
• The proposed integrated model emphasises time-invariant covariates (TICs) to predict individual differences in growth trajectories. It does not incorporate time-varying covariates (TVCs), which may offer more detailed insights into how dynamic predictors influence changes over time.

## Future work

Future research may expand upon the current framework by incorporating non-linear growth trajectories, including quadratic and piecewise models, to capture more intricate developmental patterns over time. The integration of time-varying covariates (TVCs) could also enhance the model’s ability to examine within-person changes and dynamic influences across measurement occasions. Additionally, extending the framework to multilevel or hierarchical structures may allow for more nuanced analyses of nested data commonly found in education, health, and organisational studies. Further methodological advancements could explore moderation and mediation effects within the longitudinal context, providing a richer understanding of causal mechanisms and conditional relationships over time. Applying the proposed model in various empirical domains will also help evaluate its generalizability and practical utility across diverse fields.

## Related research article

LGCM and PLS-SEM in Panel Survey Data: A Systematic Review and Bibliometric Analysis

## For a published article

Mohd Ghazali, Z., Wan Yaacob, W. F., & Wan Omar, W. M. (2023). LGCM and PLS-SEM in Panel Survey Data: A Systematic Review and Bibliometric Analysis. Data, 8(2), 32. https://doi.org/10.3390/data8020032

## Ethics statements

This study used an open-source dataset from samples collected across three waves of the Midlife Development in the United States study (1995–1996, 2004–2006, and 2011–2014)

https://www.icpsr.umich.edu/

## Supplementary material *and/or* additional information [OPTIONAL]

To facilitate replication and understanding of the proposed model, a simulated dataset and annotated R script have been made publicly available on GitHub: https://github.com/MrZMG/PLSgrowth_time-invariant_supplement

## Declaration of competing interest

The authors declare that they have no known competing financial interests or personal relationships that could have appeared to influence the work reported in this paper.

## Data Availability

Data will be made available on request.
